# Supercritical Carbon Dioxide (scCO_2_) Extraction of Phenolic Compounds from Lavender (*Lavandula angustifolia*) Flowers: A Box-Behnken Experimental Optimization

**DOI:** 10.3390/molecules24183354

**Published:** 2019-09-15

**Authors:** Katarzyna Tyśkiewicz, Marcin Konkol, Edward Rój

**Affiliations:** Supercritical Extraction Department, Sieć Badawcza Łukasiewicz - Instytut Nowych Syntez Chemicznych, Al. Tysiąclecia Państwa Polskiego 13A, 24-110 Puławy, Poland; marcin.konkol@ins.pulawy.pl (M.K.); edward.roj@ins.pulawy.pl (E.R.)

**Keywords:** lavender, optimization, phenolic compounds, supercritical fluid extraction, total phenolic content

## Abstract

Due to their numerous health benefits associated with various diseases and anti-oxidation properties, the phenolic compounds collectively referred to as phytochemicals have attracted a lot of interest, however, a single extraction method for polyphenols has not been developed yet. Supercritical fluid extraction, a green extraction method, provides the final product without organic solvent residues. In this work the extraction of lavender was performed using supercritical carbon dioxide. A statistical experimental design based on the Box-Behnken (B-B) method was planned, and the extraction yields and total phenolic contents were measured for three different variables: pressure, temperature and extraction time. The ranges were 200–300 bar, 40–60 °C and 15–45 min. The extracts yields from scCO_2_ extraction were in the range of 4.3–9.2 wt.%. The highest yield (9.2 wt.%) was achieved at a temperature of 60 °C under the pressure of 250 bar after 45 min. It also corresponded to the highest total phenolic content (10.17 mg GAE/g extract). Based on the study, the statistically generated optimal extraction conditions to obtain the highest total phenolic compounds concentration from flowers of *Lavandula angustifolia* were a temperature of 54.5 °C, pressure of 297.9 bar, and the time of 45 min. Based on the scavenging activity percentage (AA%) of scCO_2_ extracts, it is concluded that the increase of extraction pressure had a positive influence on the increase of AA% values.

## 1. Introduction

Lavender (*Lavandula angustifolia*) is a perennial plant belonging to the family of *Labiatae*, widely used for essential oil production in Europe, Northern Africa, the Middle East and Asia. Due to its properties, lavender is commonly used by various industries, for instance, as a popular aromatic shrub for perfumes. The beneficial effects of lavender are attributed to the presence of a broad range of biologically active compounds (mainly phenolic and aromatic compounds) with antioxidant, antifungal and anti-carcinogenic activities. Besides this, lavender has been proven to exhibit antimicrobial, anti-inflammatory, antidepressive and other properties. Moreover, lavender has insecticidal activity attributed to the presence of aromatic and volatile compounds, including terpenes, linalool, pinenes, etc. [1,2].

**Figure 1:**
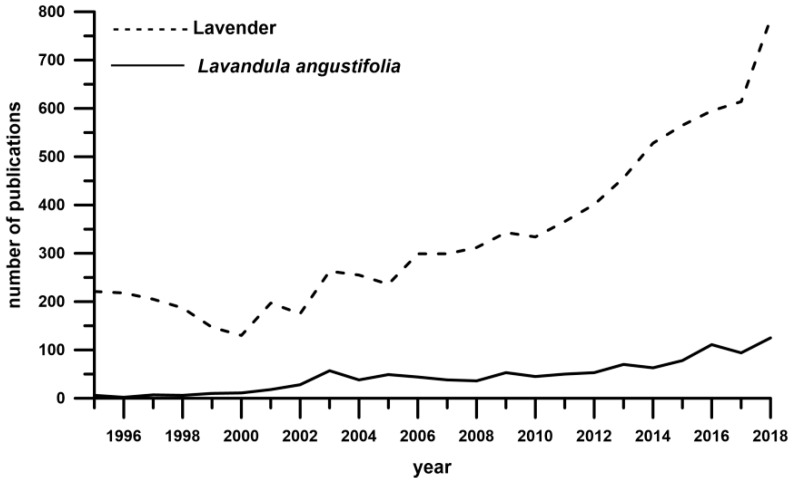
Evolution in the number of papers with the keyword “Lavender” and “*Lavandula angustifolia*” (Science Direct, April 2019).

According to Sytar et al. [3], the exact antioxidant properties of lavender are still unknown or only known to some extent. However, different studies are still being performed on lavender, especially in terms of its phenolic compounds content [4,5,6]. Figure 1 shows the increasing tendency in the interest of *Lavandula angustifolia* based on bibliographic search results with the phrase “*Lavandula angustifolia*” and “Lavender” in general.

Supercritical fluid extraction (SFE) is a separation technique, the efficiency of which depends on several aspects such as the mobile phase nature (pure or modified), the process parameters (temperature, pressure and time), and the type of raw material, as well as its pretreatment [7]. Researchers must choose and optimize the extraction parameters properly when it comes to the extraction of particular groups of compounds. However, in the case of lavender, supercritical fluid extraction is the most common method utilized for extraction of the essential oil, which is a complex matrix consisting of over one hundred compounds [3,7,8,9,10,11]. Jerković et al. [11] optimized supercritical fluid extraction of *Lavandula angustifolia* flowers in terms of the content of oxygenated monoterpenes, coumarin and herniarin. The extraction yield was influenced by the interaction between the pressure and CO_2_ flow rate, as well as between the temperature and CO_2_ flow rate. The optimal conditions based on a Box-Behnken design were w temperature of 41 °C, pressure of 100 bar and a CO_2_ flow rate of 2.3 kg/h. Similar parameters (40 °C, 100 bar) were applied by Nadalin et al. [10] for the SFE of lavender (*Lavandula officinalis*) flowers. In the study by Akgün et al. [8], the SFE was conducted at relatively low pressure (80–140 bar) and the temperature used was in the 35–50 °C range. The application of supercritical fluid extraction to phenolic compounds has been widely developed in a number of studies as gathered in a recent review by Tyśkiewicz et al. [12]. Supercritical carbon dioxide with a co-solvent was often used for the extraction of the phenolic compounds, but extraction with pure carbon dioxide was also employed [12]. In some cases, the SFE provided higher or similar extraction efficiency values compared to organic solvent (methanol, ethanol) extracts. Based on the literature reports, the methanolic and ethanolic extracts from *Baccharis dracunculifolia* were characterized by a 2-fold lower extraction yield than the scCO_2_ extract (60 °C, 400 bar) of this plant. The analysis of 3,5-diprenyl-4-hydroxycinnamic acid and 3-prenyl-4-hydroxycinnamic acid also indicated a higher content in the scCO_2_ extract [13]. However, the literature data lacks data on the optimization of *Lavandula angustifolia* extraction.

The aim of the current study was to investigate the extraction of lavender flowers using supercritical CO_2_ (green technology) to obtain polar compounds and to optimize the main extraction parameters with the use of response surface methodology (RSM) based on the extraction yield and total phenolic content.

## 2. Results and Discussion

### 2.1. Supercritical Fluid Extraction of Lavender

Supercritical fluid extraction has been applied to lavender extraction in a study by Avşar et al. [14]. *Lavandula officinalis* flowers were extracted at 45 °C and 145 bar, resulting in an extraction yield of 4.68 wt.%. Similar yield values were observed in our study, although, different parameters were required. The yields of the *Lavandula angustifolia* flower extraction were 4.3 wt.% and 4.8 wt.%, respectively, for extraction at 50 °C/300 bar and 50 °C/200 bar. Costa et al. [15] performed SFE on *Lavandula virdis* extending the extraction with two separators. In comparison with the study by Avşar et al. [14], lower extraction efficiency levels were observed (3.45 wt.% vs. 4.68 wt.%) for similar or not significantly different parameters (45 °C/145 bar vs. 40 °C/120 bar). The use of the second separator enabled achieving a higher yield of 9.27 wt.%. Increasing the pressure from 120 to 180 bar at the same extraction temperature (40 °C) did not influence the yield (3.45 wt.% vs. 3.41 wt.%). In the studies by Dahn et al. [16] volatile oils of *Lavandula angustifolia* were extracted in the range of 100–180 bar, 40–50 °C and 6.4–60 min. The highest extraction yield was obtained using the following parameters: 50 °C, 180 bar, and 60 min. The authors also succeeded in obtaining a yield of 9.0 wt.% at 53.4 °C and 140 bar. Our study resulted in obtaining the same yield (9.0 wt.%) at a higher temperature (60 °C) and over twice the pressure (300 bar). Lowering the pressure to 250 bar resulted in an even higher yield of up to 9.2 wt.%. Table 1 presents the experimental results obtained in 15 runs of *Lavandula angustifolia* flower extraction.

### 2.2. Optimization of Operating Conditions—Extraction Yield

**Figure 2:**
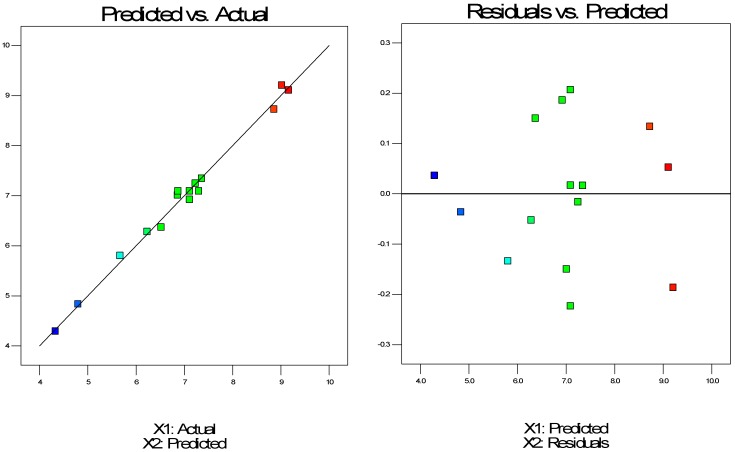
Observed extraction yield versus predicted extraction yield and the analysis of residuals.

A Box-Behnken statistical experimental design was utilized and the extraction yields were measured for different variables such as pressure, temperature and time, coded as X_1_, X_2_, X_3_. The R^2^ adjusted of the extraction yield was 97.40% indicating that chosen models allowed one to fully predict the extraction yield, as presented in Figure 2.

The response surface model (RSM) obtained from the experimental design was evaluated using ANOVA and analysis of residuals. Multiple regression coefficients were determined by applying a least-squares technique in order to predict a second-order polynomial model. Both *F*-value and *p*-value were used to determine the significance of each coefficient. Corresponding *p*-values not higher than 0.1 suggest that, among the test variables used in this study X_1_ (pressure), X_2_ (temperature), X_3_ (time), X_1_X_2_ (pressure × temperature), X_1_X_3_ (pressure × time), X_2_X_3_ (temperature × time), (X_1_)^2^ (pressure × pressure), (X_2_)^2^ (temperature × temperature), (X_3_)^2^ (time × time) are all significant model terms. The model *F*-value of 59.21 implies the model is significant. There is only a 0.02% chance that an *F*-value this large could occur due to noise. The results of variance and error for the response surface model analysis indicate that lack-of-fit is not significant relative to the pure error. There is a 49.87% chance that a lack-of-fit *F*-value this large could occur due to noise (Table 2).

**Figure 3:**
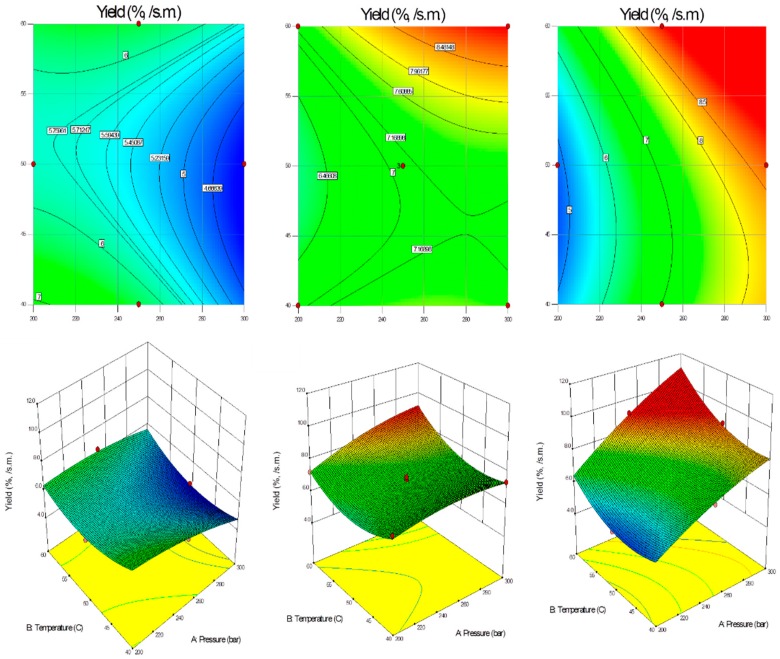
Contour plots and response surface of extraction yield as a function of supercritical CO_2_ temperature and pressure (first—15 min; middle—30 min; last—45 min).

The plots of the quadratic model with three variables kept at constant level and the other two varying within the experimental ranges are shown in Figure 3. The final equation in terms of coded variables is as follows:

(1) $$Y=7.09+0.59X_{1}+0.55X_{2}+0.87X_{3}+0.39X_{1}X_{2}+1.35X_{1}X_{3}+0.5-0.35X_{1}^{2}+0.93X_{2}^{2}-0.83X_{3}^{2}$$

The final equation in terms of actual factors is as follows:

(2) $$Y=39.1395-0.0115X_{1}-1.1704X_{2}-0.3376X_{3}+0.0008X_{1}X_{2}+0.0018X_{1}X_{3}+0.0035X_{2}X_{3}-0.0001X_{1}^{2}+0.0093X_{2}^{2}-0.0037X_{3}^{2}$$

### 2.3. Total Phenolic Content

In their studies, Sytar et al. [3] highlighted the lack of sufficient knowledge about the antioxidant activity of *Lavandula angustifolia*. Research on a methanolic extract from *Lavandula officinalis* leaves indicated the presence of phenolic compounds at the level of 2.2 mg QE/DW (quercetin/dry weight). In another study by Slavov et al. [17], SFE was applied to obtain polyphenol-rich extracts from lavender waste, which gave 7.52 mg/DW as a total phenolic content. Adaszyńska-Skwirzyńska et al. Reference [18] analyzed phenolic compounds in methanolic extracts of *Lavandula angustifolia* leaves and flowers. The contents were in the range of 3.71–4.06 mg/g DW and 1.13–1.14 mg/g DW, respectively. The flowers of *Lavandula pubescens* were characterized by a much lower content of polyphenols (234.17 μg GAE/mg) in the ethanolic extract obtained by Mousa et al. [19]. *Lavandula angustifolia* was also extracted with the use of subcritical fluid extraction, which resulted in a relatively low content of polyphenols (78.35 μg/mg total extract) in the flower extract [20]. Spiridon et al. [21] were the ones that obtained the *Lavandula angustifolia* extract with the highest level of phenolic compounds (50 mg GAE/g extract) among all performed studies described in the literature. However, methanol was used as a solvent for a whole plant extraction.

### 2.4. Optimization of Operating Conditions—Total Phenolic Content

**Figure 4:**
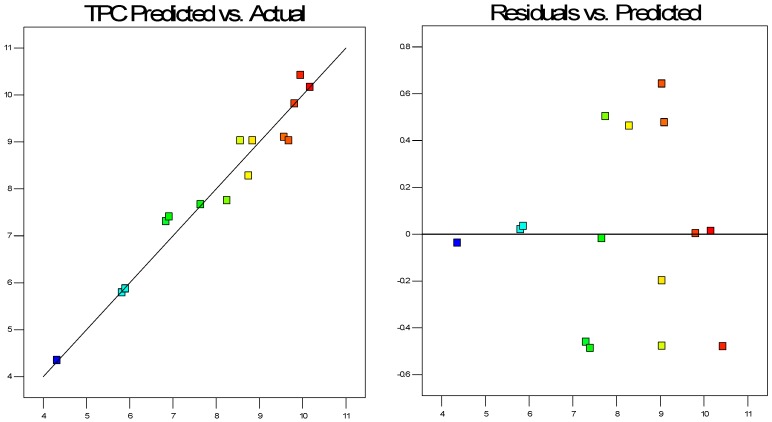
Observed total phenolic content versus predicted total phenolic content and the analysis of residuals.

A statistical experimental design based on Box-Behnken method was utilized and extraction yields were obtained for different variables such as pressure, temperature and time, coded as X_1_, X_2_, X_3_. Statistical analysis of results was performed with the use of Design Expert 9.0 (Stat-Ease, Inc., Minneapolis, MN, USA). The R^2^ adjusted of the total phenolic content was 90.65% indicating that chosen model enabled full prediction of TPC values (Figure 4).

Like for the extraction yield, ANOVA was used to evaluate the response surface model for the total phenolic content. Among the tested variables, the significant ones were X_1_ (pressure), X_3_ (time), X_1_X_2_ (pressure × temperature), X_1_X_3_ (pressure × time), X_2_X_3_ (temperature × time), (X_1_)^2^ (pressure × pressure), (X_3_)^2^ (time × time). Other variables, such as X_2_ (temperature), (X_2_)^2^ (temperature × temperature) are not significant. The model is significant, as described by the *F*-value (20.40) with a 0.04% chance that an *F*-value this large could occur due to noise. The results of a variance and error analysis for the response surface model indicate that lack-of-fit is not significant relative to the pure error. There is a 63.29% chance that a lack-of-fit *F*-value this large could occur due to noise (Table 3).

**Figure 5:**
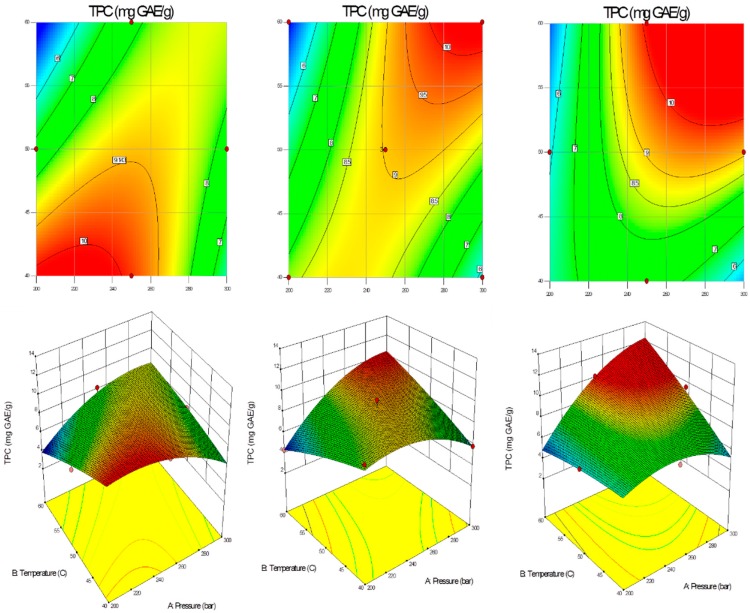
Contour plots and response surface of total phenolic content as a function of supercritical CO_2_ temperature and pressure (first—15 min; middle—30 min; last—45 min).

Figure 5 shows the effect of different operating temperature and pressure of supercritical CO_2_ on total phenolic content in the extraction time in the range of 15–45 min.

Final equation in terms of coded variables is as follows:

(3) $$\mathrm{TPC}=9.03+0.90X_{1}+0.18X_{2}+2.14X_{1}X_{2}+0.72X_{1}X_{3}+1.21X_{2}X_{3}-1.56X_{1}^{2}-0.26X_{2}^{2}$$

Final equation in terms of actual factors is as follows:

(4) $$\mathrm{TPC}=31.04792+0.086642X_{1}-1.03392X_{2}+0.004X_{1}X_{2}+0.0009X_{1}X_{3}+0.008X_{2}X_{3}-0.0006X_{1}^{2}+0.003X_{2}^{2}$$

### 2.5. Variables Affecting Procedure

The increase of the pressure to 300 bar in the 15-min-extraction resulted in a decrease of the extraction yield. On the other hand, the extraction yield was not influenced by pressure (200–300 bar) in longer extractions (30 min) at the temperature in the 40–54 °C range. Significant changes in the yield were observed above approx. 54 °C. With the extraction time of 45 min, the extraction yield increased with the increase of both pressure and temperature. The lowest extraction yield (4.3 wt.%) was observed when *Lavandula angustifolia* flowers were extracted at the temperature of 50 °C, under the pressure of 300 bar within 15 min. The highest extraction yield (9.2 wt.%) required the increase of the temperature to 60 °C as well as the decrease of the pressure to 250 bar and three times longer extraction time (45 min). One trend is observed to the yield taking into consideration parameters, especially temperature with corresponding pressure at the highest level in the experiments with a particular extraction time. In other words, the highest yield (6.5 wt.%) for the conditions with the shortest extraction time (15 min) was obtained with temperature of 60 °C and the pressure of 250 bar. The same parameters regarding temperature and pressure resulted in the highest extraction yield (9.2 wt.%) during 45 min. In the case of 30-min-extractions, the maximum parameters (60 °C, 300 bar) were the most appropriate to obtain the highest yield (9.0 wt.%) in these experiments.

Another trend was noticed as the extraction time was relevant in determining the yield but lost importance regarding total phenolic content. In the case of 15-min-extraction, the significant influence on the TPC was observed in the temperature range 40–45 °C and pressure 200–260 bar. In a comparison with the shortest extraction time, 30-min-extraction resulted in the highest TPC above approx. 54 °C and 250 bar. The relatively high TPC values were obtained also at the lowest studied temperature (40 °C) with the pressure between 220–240 bar. With the increase of the extraction time to 45 min, the decrease of TPC was noticed in the region below approx. 46 °C along studied pressure values (200–300 bar). The same temperature (60 °C) provided the lowest (4.32 mg GAE/g extract) and the highest (10.17 mg GAE/g extract) TPC values with the difference in pressure and time. As for the lowest TPC, the pressure was 200 bar and 30 min, whereas the highest total phenolic content was obtained with higher pressure (250 bar) and longer extraction time (45 min).

The scavenging activity percentage (AA%) for scCO_2_ extracts from lavender (*Lavandula angustifolia*) flowers were measured at the level of 50.55–78.83%, whereas AA% for residues was in the range of 89.88–93.44%. Among extracts, the highest antioxidant activity (78.83%) was observed for the extract obtained at 40 °C and 250 bar (15 min) and the lowest antioxidant activity (50.55%) for the extract obtained at 40 °C and lower extraction pressure (200 bar; 30 min) (Table 1). In the case of post-extraction residues, the antioxidant activity ranged 89.88–93.44% for the extraction at 60 °C, 250 bar, 15 min and 40 °C, 200 bar, 30 min. According to Dahn et al. [16], the supercritical fluid extraction of lavender (*L. angustifolia*) flowers in the pressure range 100–180 bar in terms of essential oils resulted in an antioxidant activity at the level of 32.3–73.8%. The lowest pressure (100 bar) provided the lowest AA% (32.3%), whereas the highest pressure (180 bar) was required to obtain the highest AA% (73.8%). The antioxidant activity (% inhibition of DPPH free radicals) of lavender extracts at various conditions of SFE is presented in Table 1.

**Figure 6:**
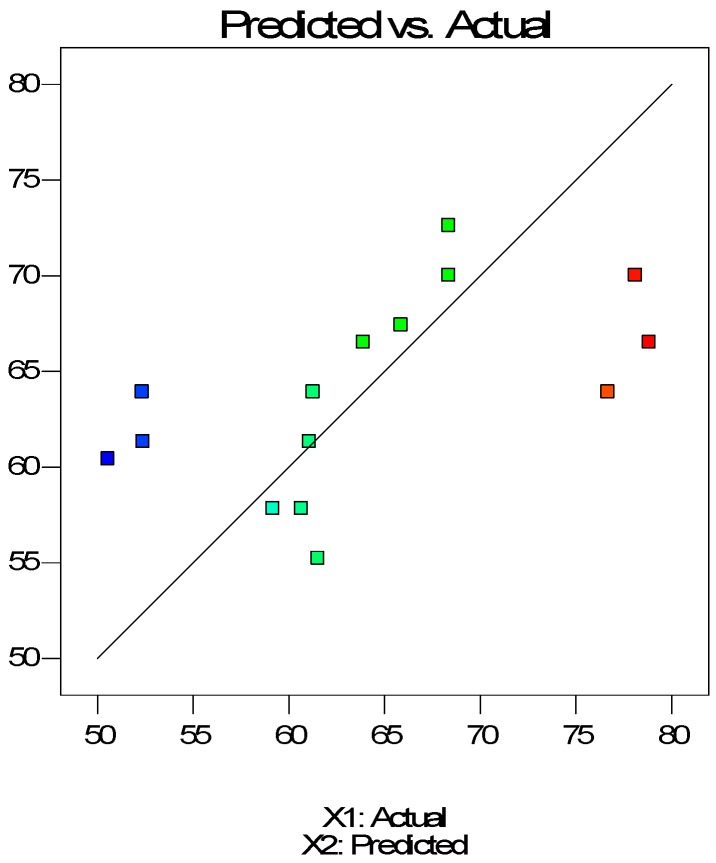
Observed antioxidant activity versus predicted antioxidant activity in extracts.

**Figure 7:**
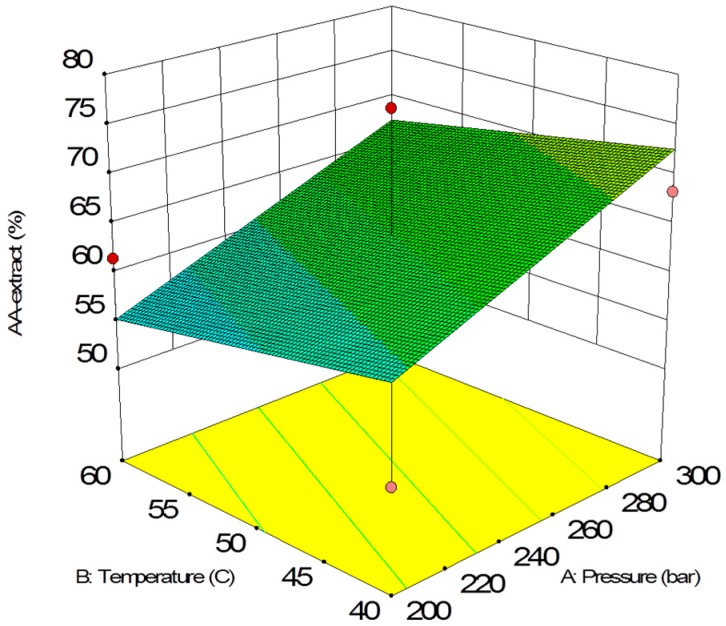
Response surface of antioxidant activity as a function of supercritical CO_2_ temperature and pressure (30 min) for extracts.

The antioxidant activity of scCO_2_ extracts is shown in Figure 6 and Figure 7 as a function of pressure and temperature with an extraction time of 30 min. In the case of extracts, pressure had a significant impact on the antioxidant activity. With the increase of pressure, AA% values also increased. On the other hand, temperature had a negative impact on AA% values.

The final equation in terms of coded variables is as follows:

(5) $$\mathrm{AA}\%=63.93+6.10X_{1}-2.60X_{2}+0.62X_{3}$$

The final equation in terms of actual factors is as follows:

(6) $$\mathrm{AA}\%=45.19110+0.12191X_{1}-0.25966X_{2}+0.041617X_{3}$$

**Figure 8:**
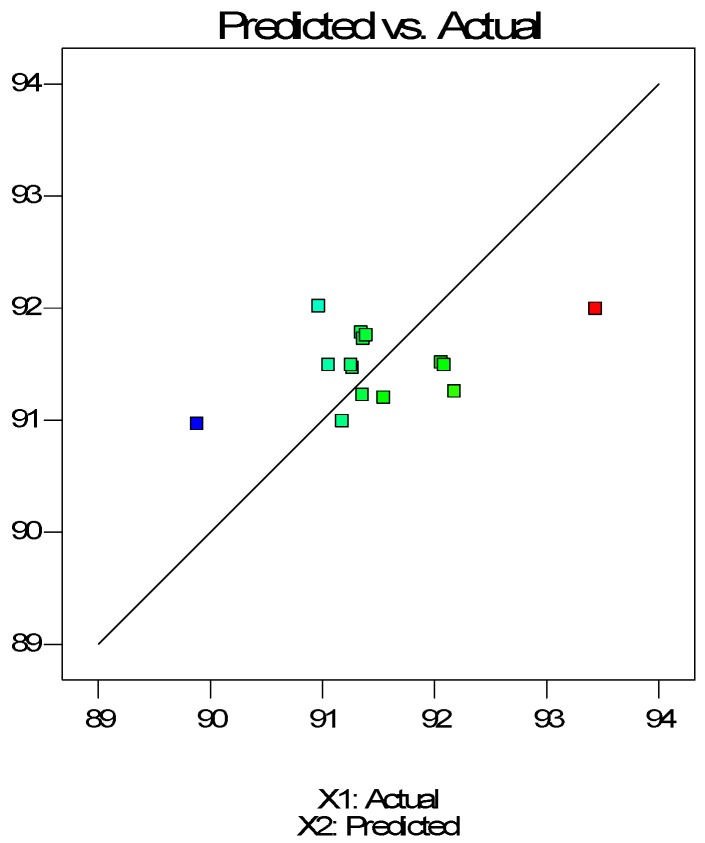
Observed antioxidant activity versus predicted antioxidant activity in residues.

**Figure 9:**
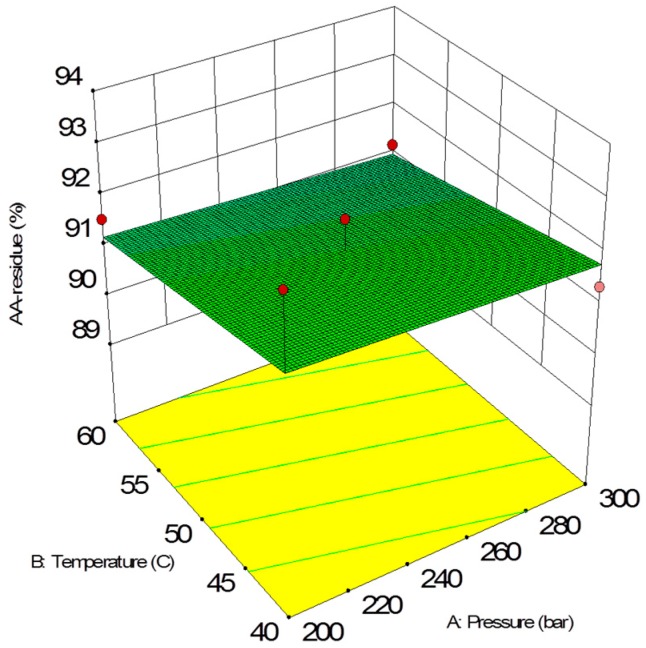
Response surface of antioxidant activity as a function of supercritical CO_2_ temperature and pressure (30 min) for residues.

The antioxidant activity of post-extraction residues is shown in Figure 8 and Figure 9 as a function of pressure and temperature with an extraction time of 30 min. In the case of residues, the observed activity values hardly depended on the pressure and temperature parameters, although, antioxidant values showed a slight downward trend as the extraction parameters increase. Time had a slight effect on the increase of AA% values.

The final equation in terms of coded variables is as follows:

(7) $$\mathrm{AA}\%=91.49-0.11X_{1}-0.40X_{2}+0.13X_{3}$$

The final equation in terms of actual factors is as follows:

(8) $$\mathrm{AA}\%=93.75031-0.0021335X_{1}-0.039659X_{2}+0.00857083X_{3}$$

## 3. The Cost of Manufacturing (COM)

For the essential oils, various extraction methods are used, as described by Rassem et al. [22], including hydrodistillation, which is a process in which both water and plant material are boiled together in an extractor. The results are distillates, also known as floral waters, hydrosols, hydrolates, herbal waters, and essential waters [23]. Steam distillation is used in large-scale production of essential oils for commercial purposes. Steam distillation uses dry steam to vaporize and extract the oil. Solvent extraction uses organic solvents to extract both essential oils and oleoresins, which are then separated. Use of many of the organic solvents would not be compatible with certified organic production. The supercritical fluid extraction method uses carbon dioxide under high pressure to extract both essential oils and oleoresins. The mixture can then be fractionated by molecular distillation or steam distillation. Supercritical extraction products belong to the “green chemistry” group [22].

### The Cost of Manufacturing Using Supercritical Fluid Extraction

Supercritical extraction is widely thought to be expensive. It is true that supercritical fluid extraction is expensive at the equipment investment stage [24]. Operating costs are however not high and are comparable to other extraction technologies. It also often happens that the cost of supercritical extraction is lower than the cost of liquid extraction. The analysis of COM dependence on the pressure (100–300 bar) and the temperature of the extraction (40–50 °C), and capacity of the extractors (0.1; 0.4 and 1.0 m^3^) is presented using Turton et al.’s formula [25]:

(9) COM=0.304FCI+2.73COL+1.23(CRM+CWT+CUT)

where FCI—fixed cost of investment, COL—cost of operational labor, CRM—cost of raw material, CWT—cost of waste treatment, CUT—cost of waste utilization (extraction residues)

Comparing the calculated COM with the real COM or market price of the same or similar product with literature data allows one to assess the analyzed project, whether it is and to what extent competitive and economically justified. This comparison also allows one to estimate possible profits. The structure of COM shows what influence on this cost particular unit costs have, which of them are crucial and which are worth looking at for savings, as well as those which have small secondary importance. The examples of COM in US$/kg of the extract are listed in Table 4.

A Rowan University group in the US compared peanut extraction with supercritical and hexane methods. The results are shown in Table 5. It is evident that the cost of extraction using CO_2_ in a supercritical state is two times lower than the cost of extraction with hexane.

## 4. Materials and Methods

The flowers of Bulgarian lavender (*Lavandula angustifolia*) were obtained from the INCO Company (Warszawa, Poland). As for the extraction process, dried flowers were milled and subjected to SFE. The size of the lavender flowers after milling (1.5 mm sieve) based on the granulometric analysis is provided in Table 6. An authentic analytical standard of gallic acid as well as Folin-Ciocalteau reagent, were purchased from Sigma-Aldrich (Darmstadt, Germany). HPLC grade ethanol (Baker), as well as anhydrous sodium carbonate (Chemsolve) were purchased from Witko (Łódź, Poland). Carbon dioxide (99.9%, *v/v*), which was used as the mobile phase in SFE, was stored in a CO_2_ installation tank.

### 4.1. Supercritical Fluid Extraction

**Figure 10:**
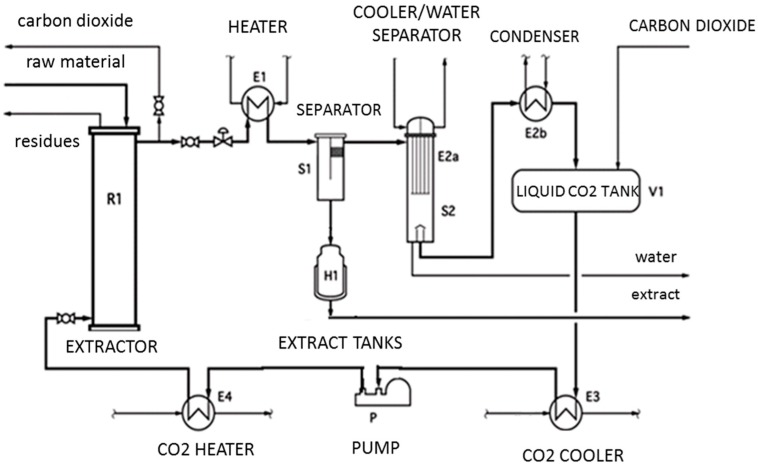
Technological scheme of the supercritical extraction unit in a lab scale.

The dynamic SFE process was performed on a laboratory scale installation, equipped with a 1 L extractor (SITEC-Sieber Engineering AG, Switzerland) operating at temperatures up to 80 °C and pressures up to 450 bar (Figure 10). Dried and milled *Lavandula angustifolia* flowers (100 g) were extracted with pure carbon dioxide. The temperature and pressure were set in the range of 40–60 °C and 200–300 bar, and the extraction time in the range of 15–45 min. The CO_2_ flow (10 kg/h) was constant for all experiments. The complete design included fifteen experiments at different conditions with the use of three center points. In order to evaluate the influence of these factors, the extraction yield (Y, % dry mass) and total phenolic content (TPC, mg GAE/g extract) were determined and used to optimize the extraction conditions. The statistical analysis of the results was performed with the Design Expert 9.0 software. Table 7 lists the ranges chosen for the three independent variables at three levels.

### 4.2. Total Phenolic Content

The uniqueness of the phenolic compounds is attributed to their over twelve studied properties, antioxidant, medical (antidiabetic, gastroprotective, cardioprotective), antibacterial, antifungal and other [30]. The nutraceutical value of lavender flowers probably results from the presence of biologically active substances from the group of coumarins, phytosterols with the main focus on essential oil components and phenolic compounds (tannins, flavonoids, anthocyanins) [31]. The literature data reports the activity of these compounds in neoplasm treatment [32]. The total phenolic content was determined spectrophotometrically using Folin-Ciocalteau reagent according to the method described in the literature [33,34]. The extracts were dissolved in ethanol (50 μL; approximately 10 mg/mL), filtered through a 0.45 μm filter and mixed with distilled water (1.58 mL) and Folin-Ciocalteu reagent (100 μL). After 30 min, an aqueous solution of sodium carbonate (300 μL) was added. After 1 h at 25 °C in the dark absorbance was measured at 765 nm using a UV-Vis spectrophotometer (V-650, Jasco, Pfungstadt, Germany). Gallic acid was used to build a calibration curve (0.1‒0.5 mg/mL; R^2^ = 0.999) and the results were expressed in milligrams of gallic acid equivalent (mg GAE/g extract). The stock solution of gallic acid (10 mg) was prepared in ethanol (2 mL). The post-extraction residues (approx. 100 mg) were extracted with 2 mL of ethanol and subjected to ultrasounds for 5 min and left for decantation. Similarly to the extracts, the residues were prepared for the reaction with Folin-Ciocalteau reagent.

### 4.3. Radical Scavenging Activity Using DPPH Method

The scavenging activity percentage (AA%) of extracts and residues was assessed by DPPH free radical assay according to the methodology described by Brand-Williams et al. [35] and Garcia et al. [36]. Each extract (approx. 50 mg) was weighed and dissolved in ethanol (1 mL). The DPPH was prepared by dissolving 19.71 mg of DPPH in 100 mL of ethanol to obtain a concentration of 0.5 mM. A volume of 0.5 mL of extract solution was transferred to a separate flask and 3 mL of ethanol and 0.3 mL of DPPH were also added. The post-extraction residues (approx. 100 mg) were extracted with 2 mL of ethanol and subjected to ultrasound for 5 min and left for decantation. The extracts from residues (0.5 mL) were filtered through a 0.45 μm filter and mixed with DPPH solution (0.3 mL) and ethanol (3 mL). The reaction mixtures were left for 30 min in the dark and the absorbance was measured at 517 nm using a UV-Vis spectrophotometer. The analyses were performed at triplicate. The percentage of inhibition was calculated according to Equation (10):

(10) $$\mathrm{AA}\%=100\left(A_{0}-A_{\mathrm{av}}\right)/A_{0}$$

where: A_0_—the absorbance of DPPH solution, A_av_—the average absorbance of samples.

## 5. Conclusions

Optimization of *Lavandula angustifolia* flowers scCO_2_ extraction shows that all three variables affect the yield of the extraction individually, as well as in combinations with each other. The effect of temperature and pressure on the extraction yield is highly pronounced. The predictive model fits well with the experimental results. The temperature, 54.5 °C; pressure, 297.9 bar; extraction time, 45 min were found to be the optimum conditions to achieve the maximum yield and maximum polyphenols content in scCO_2_ extract from *Lavandula angustifolia* flowers.

## Figures and Tables

**Table 1:** Responses of dependent variables using three levels-the factors Box-Behnken method to independent variables of the SFE method.

Exp	Pressure (bar)		Temperature (°C)		Time (min)		Observed Yield (w/wt.%)	Total Phenolic Content (mg GAE/g Extract)	Total Phenolic Content (mg GAE/g Dry Mass)	Antioxidant Activity (AA%)	
	C	U	C	U	C	U		Extracts	Residues	Extracts	Residues
1	1	300	0	50	−1	15	4.3	7.64	0.99	68.35 ± 0.7	92.18 ± 0.3
2	1	300	0	50	1	45	8.9	9.57	1.31	78.10 ± 0.4	92.06 ± 0.5
3	0	250	0	50	0	30	7.3	8.84	1.28	61.27 ± 1.1	92.09 ± 0.4
4	0	250	0	50	0	30	7.1	8.56	1.14	76.67 ± 0.4	91.05 ± 0.6
5	0	250	1	60	−1	15	6.5	8.25	1.20	52.39 ± 0.5	89.88 ± 0.9
6	−1	200	−1	40	0	30	7.1	8.75	1.32	50.55 ± 0.7	93.44 ± 1.1
7	−1	200	1	60	0	30	7.2	4.32	1.19	61.52 ± 0.8	91.55 ± 1.0
8	−1	200	0	50	−1	15	5.7	6.84	0.98	59.17 ± 0.6	91.27 ± 0.4
9	0	250	−1	40	−1	15	6.2	9.81	1.19	78.83 ± 1.3	91.39 ± 0.6
10	0	250	0	50	0	30	6.9	9.68	1.19	52.35 ± 0.1	91.25 ± 0.4
11	0	250	−1	40	1	45	6.9	6.91	1.08	63.89 ± 0.5	90.97 ± 1.2
12	−1	200	0	50	1	45	4.8	5.9	1.08	60.65 ± 0.3	91.36 ± 0.4
13	1	300	1	60	0	30	9.0	9.95	1.23	65.86 ± 0.7	91.18 ± 0.8
14	0	250	1	60	1	45	9.2	10.17	1.16	61.08 ± 0.4	91.36 ± 0.3
15	1	300	−1	40	0	30	7.4	5.82	1.30	57.00 ± 0.5	91.35 ± 0.4

C—coded, U—uncoded.

**Table 2:** Regression coefficients of predicated second-order polynomial model for the response variable.

Source	Sum of Squares	Degree of Freedom	Mean of Squares	*F*-Value	*p*-Value *
Model	26.83	9	2.98	59.21	0.0002
X_1_ (pressure)	2.83	1	2.83	56.26	0.0007
X_2_ (temperature)	2.39	1	2.39	47.42	0.0010
X_3_ (time)	6.00	1	6.00	119.26	0.0001
X_1_ × X_2_	0.59	1	0.59	11.78	0.0186
X_1_ × X_3_	7.29	1	7.29	144.82	<0.0001
X_2_ × X_3_	1.01	1	1.01	20.06	0.0065
X_1_ × X_1_	0.44	1	0.44	8.75	0.0316
X_2_ × X_2_	3.21	1	3.21	63.72	0.0005
X_3_ × X_3_	2.56	1	2.56	50.89	0.0008
Residual	0.25	5	0.050	-	-
Lack-of-fit	0.16	3	0.053	1.14	0.4987
Pure error	0.093	2	0.046	-	-

* Significant at *p* < 0.1. “-” – not applicable.

**Table 3:** Regression coefficients of predicated second-order polynomial model for the response variable.

Source	Sum of Squares	Degree of Freedom	Mean of Squares	*F*-Value	*p*-Value *
Model	41.96	7	5.99	20.40	0.0004
X_1_ (pressure)	6.43	1	6.43	21.86	0.0023
X_2_ (temperature)	0.24	1	0.24	0.83	0.3916
X_1_ × X_2_	18.32	1	18.32	62.33	<0.0001
X_1_ × X_3_	2.06	1	2.06	7.01	0.0331
X_2_ × X_3_	5.81	1	5.81	19.76	0.0030
X_1_ × X_1_	9.02	1	9.02	30.69	0.0009
X_2_ × X_2_	0.25	1	0.25	8.6	0.0384
Residual	2.06	7	0.29	-	-
Lack-of-fit	1.38	5	0.28	0.81	0.6329
Pure error	0.68	2	0.34	-	-

* Significant at *p* < 0.1. “-” – not applicable.

**Table 4:** The examples of COM in US$/kg of the extract [26,27,28].

Extract	COM (US$/kg Extract)
Clove (*Dianthus*)	9.15
Cloves	4.70
Lavender Retail value of lavender oil	66.50 2400 $/dm^3^
Ginger	99.80
Buriti palm	22.81
Pupunha palm	17.15
Pomegranate leaves	114.36
Habanero pepper	540.19

**Table 5:** Comparison of the extraction cost of peanut with carbon dioxide and hexane [29].

Factor	Carbon Dioxide Extraction	Hexane Extraction
Solvent	0.07 $/lb	0.07 $/lb
Max solubility	38 mg/g	80 mg/g
CO_2_ flow	87 million lb/yr	38 million lb/yr
Energy input	1.8 GWh/y	4.6 GWh/yr
COM	6.2 million $/yr	14 million $/yr

**Table 6:** The size of milled lavender flowers for the SFE.

Fraction Size (mm)	Fraction Mass (g)	Fraction Percentage (%)
2.50	0.01	0.01
2.00	0.08	0.10
1.60	0.11	0.14
1.00	6.22	7.93
0.80	7.32	9.33
0.60	17.26	21.99
0.40	16.68	21.25
0.30	11.05	14.08
0.10	17.82	22.71
<0.1	1.93	2.46
SUM	78.48	100

**Table 7:** Ranges of three independent variables in the Box-Behnken method.

Variables	Unit	−1	0	1
Temperature	°C	40	50	60
Pressure	bar	200	250	300
Time	min	15	30	45

## References

[1] Zheljazkov V.D., Cantrell C.L., Astatkie T., Jeliazkova E. (2013). Distillation time effect on lavender essential oil yield and composition. J. Oleo Sci..

[2] Cavanagh H.M.A., Wilkinson J.M. (2002). Biological activities of lavender essential oil. Phytother. Res..

[3] Sytar O., Hemmerich I., Zivcak M., Rauh C., Brestic M. (2018). Comparative analysis of bioactive phenolic compounds composition from 26 medicinal plants. Saudi J. Biol. Sci..

[4] Yadikar N., Bobakulov K., Li G., Aisa H.A. (2018). Seven new phenolic compounds from *Lavandula angustifolia*. Phytochem. Lett..

[5] Zhao J., Xu F., Huang H., Ji T., Li C., Tan W., Chen Y., Ma L. (2015). Evaluation of bioactivities of total flavonoids from *Lavandula angustifolia*. Pak. J. Pharm. Sci..

[6] Wu X., Liu J., Yu Z., Ye Y., Zhou Y. (2007). Chemical constituents of *Lavandula augustifolia*. Acta Chim. Sinica.

[7] Adasoglu N., Dincer S., Bolat E. (1994). Supercritical-fluid extraction of essential oil from Turkish Lavender flowers. J. Supercrit. Fluids..

[8] Akgün M., Akgün N.A., Dincer S. (2000). Extraction and modeling of Lavender flower essential oil using supercritical carbon dioxide. Ind. Eng. Chem. Res..

[9] Capuzzo A., Maffei M.E., Occhipinti A. (2013). Supercritical fluid extraction of plant flavors and fragrances. Molecules.

[10] Nadalin V., Lepojvić Z., Ristić M., Vladić J., Nikolovski B., Adamović D. (2014). Investigation of cultivated Lavender (*Lavandula officinalis* L.) extraction and its extract. Chem. Ind. Chem. Eng..

[11] Jerković I., Molnar M., Vidović S., Vladić J., Jokić S. (2017). Supercritical CO_2_ extraction of Lavandula angustifolia Mill. Flowers: Optimisation of oxygenated monoterpenes, coumarin and nerniarin content. Phytochem. Anal..

[12] Tyśkiewicz K., Konkol M., Rój E. (2018). Application of supercritical fluid extraction in phenolic compounds isolation from natural plant materials. Molecules.

[13] Piano C.R., Aquino F.W.B., Follegatti-Romero L.A., Cabral F.A. (2008). Supercritical CO_2_ extraction compounds from Baccharis dracunculifolia. J. Supercrit. Fluids.

[14] Avşar G., Yüksel D., Emen F.M., Demirdöğen R.E., Yeşilkaynak T., Kahriman L. (2018). Supercritical carbon dioxide extraction of Lavandula officinalis (lavender) and Hypericum perforatum (centaury) plants grown in mersin region: Investigation of antioxidant and antibacterial activities of extracts and usage as cosmetic preservatives in creams. J. Turkish Chem. Soc..

[15] Costa P., Grosso C., Gonçalves S., Andrade P.B., Valentão P., Bernardo-Gil M.G., Romano A. (2012). Supercritical fluid extraction and hydrodistillation for the recovery of bioactive compounds from *Lavandula virdis* L’Her. Food Chem..

[16] Dahn N.T., Triet N.D.A., Han L.T.N., Zhao J., Mammucari R., Foster N. (2012). Antioxidant activity, yield and chemical composition of lavender essential oil extracted by supercritical CO_2_. J. Supercrit. Fluid..

[17] Slavov A.M., Karneva K.B., Vasileva I.N., Denev P.N., Denkova R.S., Shikov V.T., Manolova M.N., Lazarova Y.L., Ivanona V.N. (2018). Valorization of lavender waste—obtaining and characteristics of polyphenol rich extracts. Food Sci. App. Biotechnol..

[18] Adaszyńska-Skwirzyńska M., Dzięcioł M. (2017). Comparison of phenolic acids and flavonoids contents in various cultivars and parts of common lavender (*Lavandula angustifolia*) derived from Poland. Nat.Prod. Res..

[19] Mousa O., Gouda B., Salama M., El-Eraky W., Kassem H. (2018). Total phenolic, total flavonoid content, two isolates and bioactivity of *Lavandula pubescens* Decne. Int. J.Pharmacogn. Phytochem. Res..

[20] Radulescu C., Stihi C., Ilie M., Lazurcă D., Gruia R., Olaru O.T., Bute O., Dulama I.D., Stirbescu R.M., Teodorescu S. (2017). Characterization of phenolic in *Lavandula angustifolia*. Anal. Lett..

[21] Spiridon I., Bodirlau R., Teaca C.A. (2011). Total phenolic content and antioxidant activity of plants used in traditional Romanian herbal medicine. Cent. Eur. J. Biol..

[22] Hesham H.A., Abdurahman H.N., Rosli M.Y. (2016). Techniques for extraction of essential oils from plants: A review. Aust. J. Basic Appl. Sci..

[23] Edris A.E. (2009). Identification and absolute quantification of the major water-soluble aroma components isolated from the hydrosols of some aromatic plants. J. Essent. Oil Bear. Plants.

[24] Prado J.M., Assis A.R., Maróstica-Júnior M.R., Meireles M.A.A. (2010). Manufacturing cost of supercritical-extracted oils and carotenoids from Amazonian plants. J. Food Process. Eng..

[25] Turton R., Bailie R.C., Whiting W.B., Shaeiwitz J.A. (1998). Analysis, Synthesis and Design of Chemical Process, PTR.

[26] Rosa P.T.V., Meireless M.A.A. (2005). Rapid estimation of the manufacturing cost of extracts obtained by supercritical fluid extraction. J. Food Eng..

[27] Rocha-Uribe J.A., Novelo-Pérez J.I., Ruiz-Mercado C.A. (2014). Cost estimation for CO_2_ supercritical extraction systems and manufacturing cost for habanero chili. J. Supercrit. Fluids.

[28] Adam K.L. Lavender Production, Markets, and Agritourism. http://www.attra.ncat.org.

[29] Gifford M., Biancani E., Kearsley W., Maluchnik W., Farrell S., Savelski M.J., Hesketh R.P. Economic Feasibility Study on the Supercritical Fluid Extraction of Edible Oils.

[30] Veggi P.C., Prado J.M., Bataglion G.A., Eberlin M.N., Meireles M.A.A. (2014). Obtaining phenolic compounds from jatoba (Hymenaea courbaril L.) bark by supercritical fluids extraction. J. Supercrit. Fluids.

[31] Nurzyńska-Wierdlak R., Zawiślak G. (2015). Chemical composition and antioxidant activity of lavender (Lavandula angustifolia) aboveground parts. Acta Sci. Pol. Hortorum Cultus.

[32] Soobrattee M.A., Bahorun T., Aruoma O.I. (2006). Chemopreventive action of polyphenolics compounds in cancer. Biofactors.

[33] Waterhouse A.L. (2002). Determination of total phenolics. Curr. Protocols Food Anal. Chem..

[34] Makanjuola S.A. (2017). Influence of particle size and extraction solvent on antioxidant properties of extracts of tea, ginger, and tea-ginger blend. Food Sci. Nutr..

[35] Brand-Wiliams W., Cuvelier M.E., Berset C. (1995). Use of a free radical method to evaluate antioxidant activity. Lebenson Wiss Technol..

[36] Garcia E.J., Oldoni T.L.C., Reis A., Loguercio A.D., Grande R.H.M. (2012). Antioxidant activity by DPPH assay of potential solutions to be applied on bleached teeth. Braz. Dent. J..

